# The Natural Anticancer Agent Plumbagin Induces Potent Cytotoxicity in MCF-7 Human Breast Cancer Cells by Inhibiting a PI-5 Kinase for ROS Generation

**DOI:** 10.1371/journal.pone.0045023

**Published:** 2012-09-13

**Authors:** Ju-Hee Lee, Ji-Hyun Yeon, Hanna Kim, Whijae Roh, Jeiwook Chae, Han-Oh Park, Dong-Myung Kim

**Affiliations:** *S. pombe* Research Team, Gene-to-Drug Division, Bioneer Corporation, Daejeon, Republic of Korea; Wayne State University School of Medicine, United States of America

## Abstract

Drug-induced haploinsufficiency (DIH) in yeast has been considered a valuable tool for drug target identification. A plant metabolite, plumbagin, has potent anticancer activity via reactive oxygen species (ROS) generation. However, the detailed molecular targets of plumbagin for ROS generation are not understood. Here, using DIH and heterozygous deletion mutants of the fission yeast *Schizosaccharomyces pombe*, we identified 1, 4-phopshatidylinositol 5-kinase (PI5K) its3 as a new molecular target of plumbagin for ROS generation. Plumbagin showed potent anti-proliferative activity (GI_50_; 10 µM) and induced cell elongation and septum formation in wild-type *S. pombe*. Furthermore, plumbagin dramatically increased the intracellular ROS level, and pretreatment with the ROS scavenger, N-acetyl cysteine (NAC), protected against growth inhibition by plumbagin, suggesting that ROS play a crucial role in the anti-proliferative activity in *S. pombe*. Interestingly, significant DIH was observed in an its3-deleted heterozygous mutant, in which ROS generation by plumbagin was higher than that in wild-type cells, implying that its3 contributes to ROS generation by plumbagin in this yeast. In MCF7 human breast cancer cells, plumbagin significantly decreased the level of a human ortholog, 1, 4-phopshatidylinositol 5-kinase (PI5K)-1B, of yeast its3, and knockdown of PI5K-1B using siPI5K-1B increased the ROS level and decreased cell viability. Taken together, these results clearly show that PI5K-1B plays a crucial role in ROS generation as a new molecular target of plumbagin. Moreover, drug target screening using DIH in *S. pombe* deletion mutants is a valuable tool for identifying molecular targets of anticancer agents.

## Introduction

The plant metabolite, plumbagin (5-hydroxy-2 methyl-1,5-naphthoquinone), is a naphthoquinone derivative that was originally identified from the roots of plant Plumbago and belongs to one of the largest and diverse groups of plant metabolites [1], [2], [3]. Plumbagin has potent anti-proliferative and apoptotic activities in various types of human cancers, but the mechanisms underlying the anticancer activity are only partially understood. This compound dysregulates multiple pathways that play a crucial role in cancer cell proliferation, survival, invasion and metastasis [4], [5], [6], [7], [8], [9], in which ROS generation is a critical mediator for cell cycle arrest and apoptosis [6], [10], [11]. However, molecular insights for ROS generation by this agent are not clearly defined.

Phosphatidylinositol lipids have been implicated in various cellular events such as cell survival, mitogenesis, and morphological changes [12]. A number of phosphatidylinositol kinases (PIKs) are responsible for the activation of these lipids through the phosphorylation of the inositol ring. Phosphatidylinositol-4, 5-bisphosphate 3-kinase (PI3K) is the most well-characterized PIK and has a functional role in development of cancers; thus, PI3K has been a therapeutic target for anticancer agents [13]. Interestingly, PI3K as well as NF-κB and Bcl2 were reported to be a molecular target of plumbagin in human breast cancer cells–plumbagin dramatically decreased the level of the PI3K subunit p85, thereby inhibiting the downstream Akt/mTor pathway leading to growth arrest and cell death [14], [15]. 1, 4-phopshatidylinositol 5-kinase (PI5K) is another type of kinase that phosphorylates the 5-carbon of the inositol ring of 1, 4-phopshatidylinositol. This kinase regulates cell morphology and the endosomal pathway in mammalian cells as well as cell integrity and cytokinesis in the fission yeast *Schizosaccharomyces pombe* [16], [17], [18]. However, the functional role of PI5K as a molecular target of plumbagin is not known.

Drug-induced haploinsufficiency (DIH) in yeast is a powerful tool for drug target identification, and a number of reports have already shown successful drug target identifications using DIH in the budding yeast *Saccharomyces cerevisiae*. As a model organism of mammalian cells, *S. pombe* is considered superior to *S. cerevisiae* because its cell division pattern is similar to that of mammalian cells. Here, using our fission yeast *S. pombe* heterozygous deletion mutant library [19] and a high-throughput genome-wide drug target identification service system (GPScreen™) incorporating DIH in *S. pombe* genome-wide heterozygous deletion mutants (http://www.bioneer.co.kr/products/GPScreen/GPScreen-overview.aspx), we identified a 1, 4-phopshatidylinositol 5-kinase (PI5K) its3 as a new molecular target of plumbagin and defined the functional role of the target in ROS generation by this agent. In this study, plumbagin showed a potent anti-proliferative activity in *S. pombe* in an ROS-dependent manner, which was very similar to the patterns in human cancer cells. Interestingly, prominent DIH was observed in an its3-deleted heterozygous mutant. Notably, ROS generation by plumbagin in the mutant was also more potent and prolonged compared to that of wild-type cells. Furthermore, in human breast cancer MCF-7 cells, plumbagin dramatically decreased the level of PI5K-1B, which is a human ortholog of yeast its3, and knockdown of PI5K-1B using a PI5K-1B-specific siRNA significantly inhibited cancer cell viability. Taken together, these data indicate that PI5K-1B might be a new molecular target of plumbagin and play a crucial role in ROS generation for the cytotoxicity by this agent, and drug target screening using DIH in an *S. pombe* heterozygous deletion mutant library is a valuable tool for both drug target identification and mode-of-action studies of drug candidates for improving the success rate of drug discovery.

## Materials and Methods

### Materials

Plumbagin, sulforhodamine B, paraformaldehyde solution, N-acetyl-cysteine (NAC), and rabbit polyclonal antibodies against β-actin and PI-5 kinase 1B were purchased from Sigma Chemical Co. (St. Louis, MO, USA). Antibody against PI3K p85 (rabbit polyclonal) was from Abcam (Cambridge, MA, USA). Dihydroethidium (DHE) was obtained from Invitrogen Molecular Probes (Eugene, OR, USA). Anti-mouse and anti-rabbit horseradish peroxidase–linked secondary antibodies were purchased from Amersham Pharmacia Biotech (Uppsala, Sweden) and Bio-Rad (Hercules, CA, USA), respectively. ECL chemiluminescence reagent was from Millipore (Bedford, MA, USA). Lipofectamine LTX reagent was from Invitrogen (Carlsbad, CA, USA). WST-1 reagent and protease inhibitor cocktail were purchased from Roche (Nutley, NJ, USA).

### *S. pombe* Heterozygous Deletion Mutant Strains

All *S. pombe* strains including wild-type (SP286; h+/h+, ade6-M210/ade6-M216, ura4-D18/ura4-D18, leu1-32/leu1-32) and heterozygous deletion mutants were obtained from Bioneer (Daejeon, Korea). *S. pombe* heterozygous deletion mutants were constructed as described [19].

### *S. pombe* Cell Culture and Drug-induced Haploinsufficiency Analysis

*S. pombe* cells were cultured at 30°C in complete YES media containing 0.5% yeast extract, 2% glucose and various supplements as described [20]. DIH by plumbagin was measured with a cell proliferation assay as follows. Cells were diluted to a density of 1×10^6^ cells/ml in YES medium, and 50 µl was seeded into each well of 96-well plates. Then, cells were treated with 50 µl of plumbagin in EMM medium such that the final plumbagin concentration was 0.1–100 µM. Cultures were incubated at 30°C, and OD_600_ was recorded every 2 h for 14 h using a microplate reader (TECAN; M200Pro). The GI_50_ value was determined by analyzing the concentration of the compound for 50% inhibition of cell growth compared with control. Morphological changes in the presence of plumbagin were also observed via microscopy (Olympus; BX53), and growth inhibition by plumbagin was confirmed alternatively using a spot assay in agar plates. For the spot assay, cells treated without or with 10 µM of plumbagin were serially four-fold diluted, spotted onto YES agar plates, and incubated for 3 days when the colonies were appeared. Then, the size of each colony was compared.

### Cancer Cell Culture

All tumor cell lines including MCF7 human breast cancer cells were maintained in RPMI 1640 containing 5% (v/v) fetal bovine serum 1× antibiotics (100 U/ml penicillin, 100 µg/ml streptomycin). Cells were maintained at 37°C under 5% CO_2_.

### Cell Viability Assay (WST-1 Assay)

The WST-1 assay was performed to measure cell viability according to the manufacturer’s instructions (Roche). Briefly, 10 µl of WST-1 reagent was added to 100 µl of cell culture in each well of 96-well plates and incubated for 3–4 h in the dark. Then, OD_440_ was measured to determine cell viability after plumbagin treatments.

### Western Blot Analysis

After each treatment, cells were washed twice with PBS and harvested in PBS containing a protease inhibitor cocktail (Roche; 1 tablet/50 ml PBS). Cells were then lysed by sonication on ice, and whole-cell extracts were centrifuged at 10,000× *g* for 15 min at 4°C, and the protein concentration in each supernatant was measured using the Bradford method; proteins were then analyzed by western blotting.

### Fluorescence Microscopy for ROS Analysis

After each treatment with plumbagin, live cell labeling of the treated cells with 4 µM DHE were performed for 30 min in culture conditions, followed by fixation of the cells with 4% para-formaldehyde in PBS for 15 min at room temperature. Then, they were washed with PBS five times and analyzed by either fluorescence microscopy or in a fluorescence microplate reader (TECAN; M200Pro, excitation 518 nm, emission 605 nm).

### RT-PCR

To determine the levels of PI5K-1B and GAPDH mRNAs in human cancer cells, RT-PCR analysis was performed according to the following procedures. Total RNA was purified from siRNA-treated cancer cells using the Qiagen RNeasy Mini kit (Hilden, Germany) according to the manufacturer’s guidance. RT-PCR amplification of total RNA (200 ng/reaction) was performed using PI5K-1B-specific primers (forward, 5′-CCAGCACATCACTACCCAGAC-3′; reverse, 5′-ACTGGCTCCAGGGTTAGACAG-3′) and GAPDH-specific primers (forward, 5′- GGTGAAGGTCGGAGTCAACG-3′; reverse, 5′-ACCATGTAGTTGAGGTCAATGAAGG-3′) in a 20- µl reaction using the Accupower RocketScript Cycle RT PreMix and Accupower Hotstart PCR PreMix (Bioneer, Taejeon, Korea). PCR amplification (500 ng cDNA/reaction) was performed in a 20- µl reaction as follows: one cycle at 50°C for 1 h, 94°C for 5 min, followed by 32 cycles at 94°C for 30 s, 58°C for 30 s, and 72°C for 1 min, with one final extension step at 72°C for 5 min. PCR reaction products were analyzed by agarose gel electrophoresis.

### siRNA Study

MCF-7 cells (4.0×10^5^) were seeded into each 6-well plate with 2.5 ml of culture medium without antibiotics one day before transfection so that they would be 50–60% confluent at the time of transfection. The next day, siRNA transfection was performed using Lipofectamine RNAiMAX after exchanging the culture medium in each well with 500 µl fresh medium lacking serum. Briefly, siRNA duplex-Lipofectamine RNAiMAX complexes were prepared using either PI5K-1B-specific siRNA (5′-GUCCUCAAUUAGCCAGGAAdTdT-3′) or a control siRNA (5′-CUUACGCUGAGUACUUCGAdTdT-3′) and transfected into cancer cells according to manufacturer’s instructions (each primer, 20 nM final concentration) in 1.0 ml of Opti-MEM I without serum. After incubation for 5–6 h, the media were exchanged with fresh media containing serum, and cells were incubated for 48 h for gene knockdown.

### Statistical Analysis

Data are presented as the mean±S.D. Statistic analysis was performed using Student’s *t*-test for comparisons between two independent samples.

## Results

### Plumbagin Induces Potent Cytotoxicity in MCF-7 Human Breast Cancer Cells in an ROS-dependent Manner

**Figure 1:**
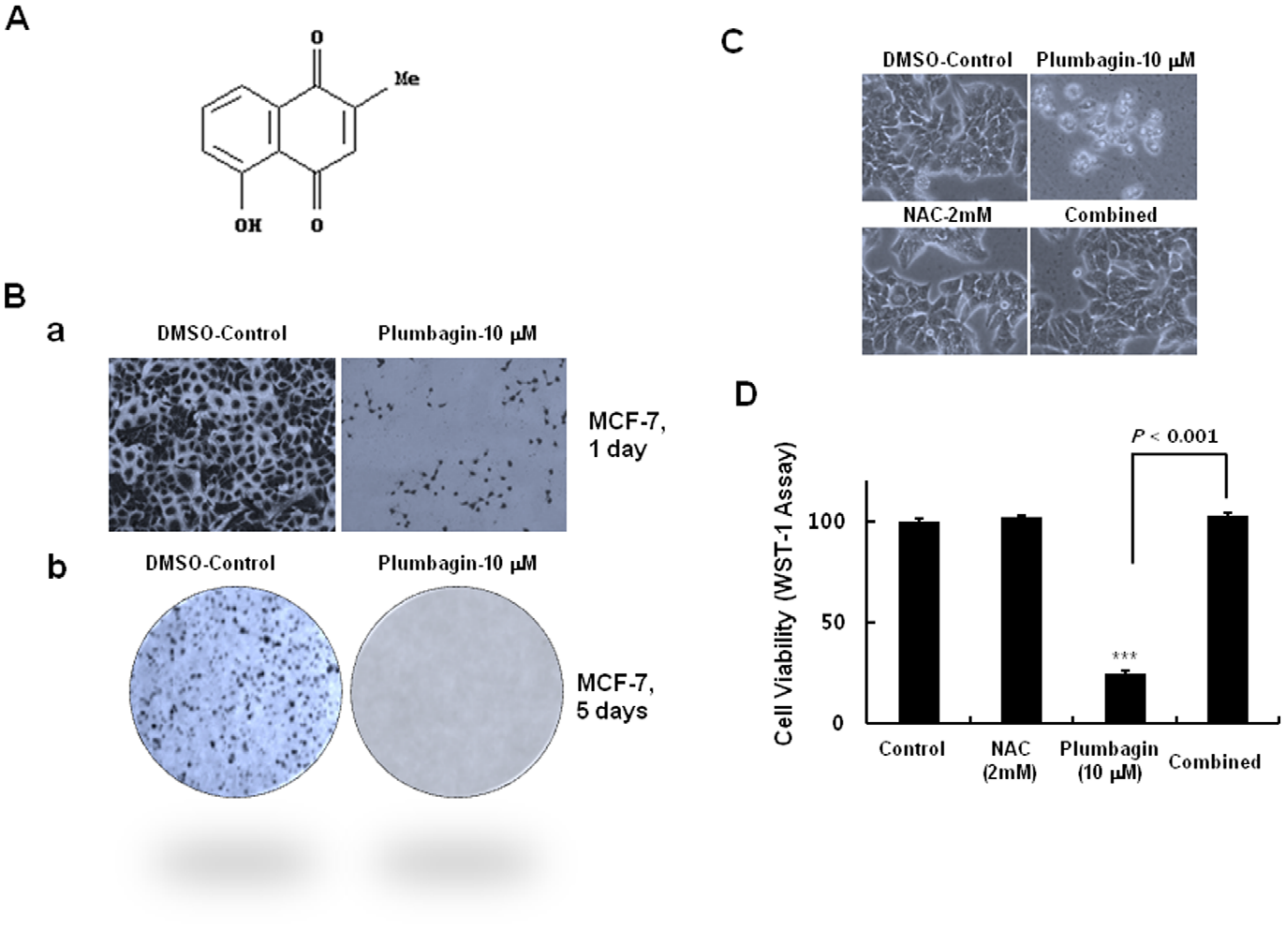
Cytotoxic effects of plumbagin on MCF-7 human breast cancer cells in an ROS-dependent manner. (A) Chemical structure of plumbagin. (B) Cells were plated onto 6-well plates at 2×10^4^ cells/well of 96-well plate. The following day, cells were treated with 10 µM of plumbagin, and incubated for 24 h. Then, Cells were then stained using the SRB and photographed (Ba). MCF-7 cells were plated onto 6-well plates at 2×10^4^ cells/well. The following day, cells were treated with 10 µM, and incubated for 5 days. Then, colony formations were observed (Bb). (C) The viability of the treated cells in cells were treated with 10 µM of plumbagin in the presence or absence of 2 mM NAC, a ROS scavenger, and incubated for 24 h. Then, cells were photographed. (D) The viability of the treated cells in (B) was measured with the WST-1 assay. Data represent the mean ± standard error (n = 3). ***P<0.001 compared with the untreated samples.

ROS are critical mediators of plumbagin-induced toxicity in human cancer cells [6], [10], [21], [22]. However, the detailed mechanisms of ROS generation by plumbagin (Figure 1A) are not defined. Therefore, we tried to understand the molecular insights for ROS generation. We first confirmed the functional role of ROS in MCF-7 cells because this cell line was most sensitive to this agent among various types of human cancer cells tested in our study (Figure S1 &S2). Treatment with 10 µM plumbagin to MCF-7 cells induced potent cytotoxicity in sulforhodamine B (SRB) assay for 24 h (Figure 1Ba), which correlated well with a previous report [14] and dramatically inhibited colony formations of the treated cells for 5 days (Figure 1Bb). The treated cells were shrunken, detached from the culture plates, and had acquired a round shape, indicating that the cells were dying (Figure 1C, upper lane). However, prior treatment with 2 mM NAC, an ROS scavenger, blocked most of the plumbagin-induced cell death (Figure 1C, bottom lane), which was revealed alternatively using cell viability analysis using WST-1 reagent (Figure 1D), supporting that ROS plays a crucial role in plumbagin-induced death of MCF-7 cells. These patterns were also observed in other cancer cells such as HeLa human cervical cancer cells and A549 human lung carcinoma cells (Figure S3). These results indicated that ROS are critical mediators of plumbagin cytotoxicity on human cancers and that defining the molecular insights of ROS generation by plumbagin might provide therapeutic benefits for drug discovery as well as understanding its mode of action.

### Plumbagin Potently Inhibits Growth of Wild-type *S. pombe* in an ROS-dependent Manner

**Figure 2:**
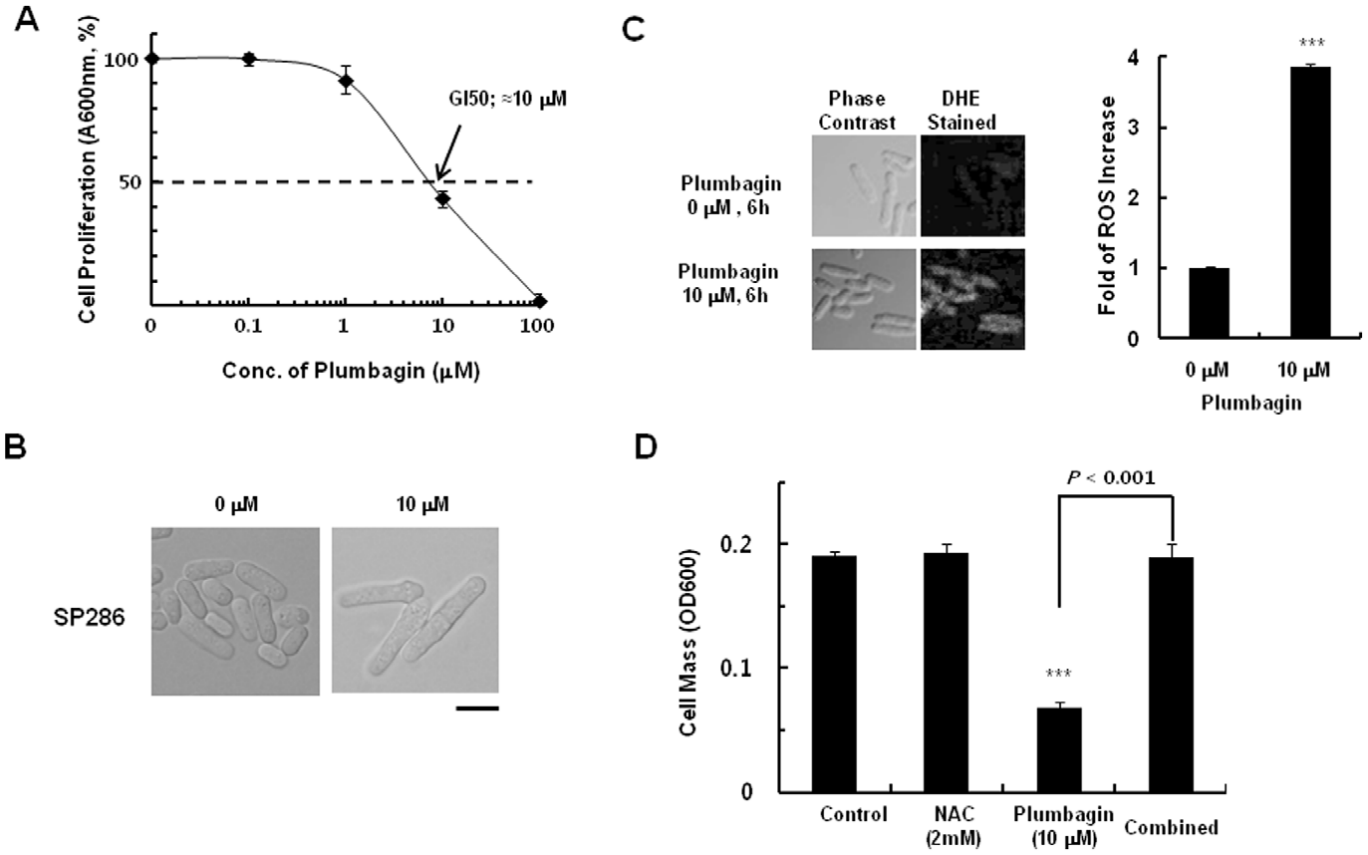
ROS-dependent growth inhibition of wild-type *S. pombe* (SP286) by plumbagin. (A) Wild-type *S. pombe* cells were plated onto 96-well plates at 1×10^5^ cells/well, followed by treatment with various concentrations of plumbagin and incubation at 30°C for 14 h. Then, OD_600_ was measured using a microplate reader. Data represent the mean ± standard error (n = 3). (B) *S. pombe* cells were left untreated or were treated with 10 µM plumbagin and incubated for 6 h. Then, cells were photographed. The scale bar below the right figure represents 10 µm. (C) Cells were treated with 0 or 10 µM plumbagin for 6 h and were stained with 4 µM DHE, an ROS staining reagent, for 30 min. Then, the level of ROS was measured by fluorescence microscopy as indicated in Materials and Methods. (D) Wild-type *S. pombe* cells were treated with 10 µM plumbagin in the absence or presence of NAC (2 mM) for 14 h, then the cell mass was measured as OD_600_ in a microplate reader. Data represent the mean ± standard error (n = 3). ***P<0.001 compared with the untreated samples.

Next, we tried to define the intracellular molecular targets of plumbagin using DIH in *S. pombe* heterozygous deletion mutants aiming at understanding the molecular insights of ROS generation by the agent. We first investigated the growth-inhibitory activity of plumbagin in wild-type *S. pombe* (SP286). In the treatment to wild-type *S. pombe* cells for 14 h, plumbagin showed potent anti-proliferative activity (GI_50_ = 10 µM; Figure 2A). Furthermore, it induced cell elongation and septum formation (Figure 2B), representative of G2-M arrest as demonstrated in mammalian cells [14]. Notably, plumbagin induced a dramatic increase in ROS generation as early as 6 h, which was revealed by DHE staining (Figure 2C & Figure S4). Moreover, prior treatment with 2 mM NAC protected the growth inhibition of SP286 by plumbagin dramatically (Figure 2D), which was very similar to the effects in cancer cells. These results indicated that plumbagin potently inhibits the growth of wild-type *S. pombe* cells in an ROS-dependent manner.

### Plumbagin Induces Drug-induced Haploinsufficiency in its3-deleted Heterozygous Mutants of *S. pombe*

**Figure 3:**
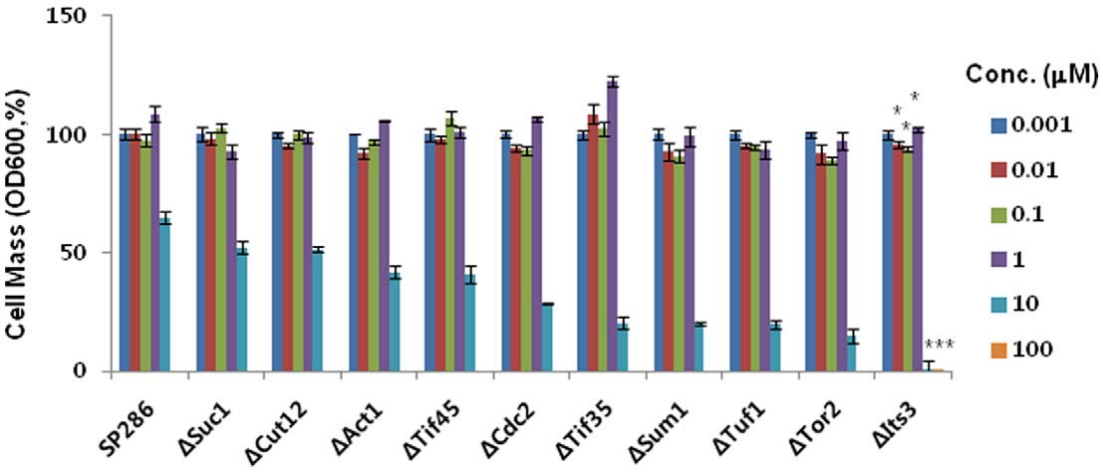
Significant plumbagin-induced haploinsufficiency in its3-deleted a heterozygous mutant among various mutants. Wild-type *S. pombe* (SP286) and each heterozygous mutant were diluted to an OD_600_ of 0.1, and 50 µl of each diluted sample was seeded into another 96-well plate. Then, cells in culture were treated with 50 µl of 2× concentrations of plumbagin to be tested in culture media as described in the “Materials and Methods”. Cultures were incubated at 30°C for 14 h, and OD_600_ was measured using a microplate reader. Data represent the mean ± standard error (n = 3).

**Figure 4:**
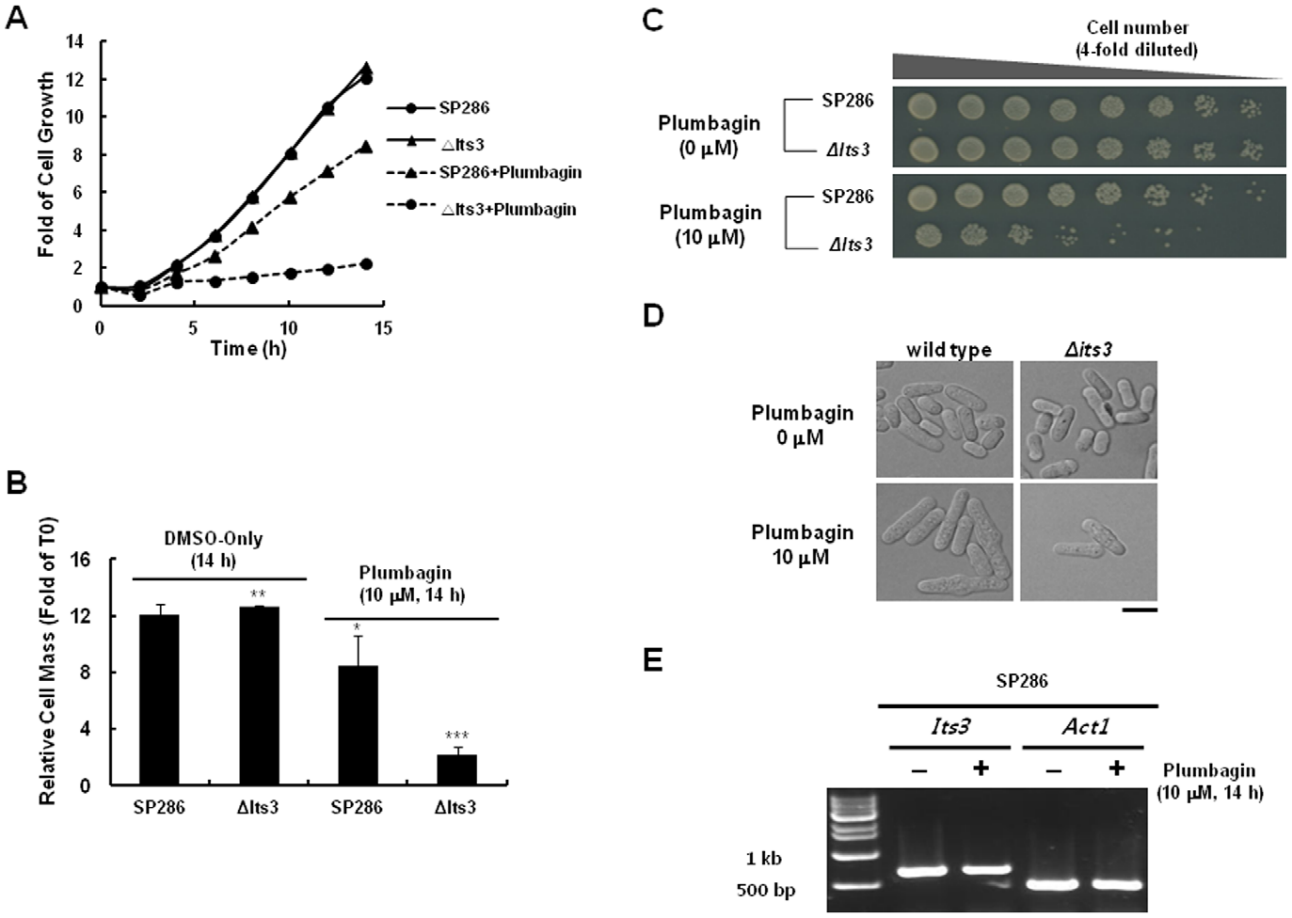
Induction of drug-induced haploinsufficiency by plumbagin in an its3-deleted heterozygous mutant. (A) Wild-type *S. pombe* (SP286) and an its3-deleted heterozygous mutant (SPAC19G12.14; its3::KanMX) were treated with 10 µM plumbagin under the same conditions as in Figure 3. Cultures were incubated at 30°C, and OD_600_ was recorded every 2 h for 14 h using a microplate reader. (B) Relative cell mass at 14 h after the treatment in (A) was measured as OD_600_. Data represent the mean ± standard error (n = 3). *P<0.05, **P<0.01, ***P<0.001 compared with the untreated samples. Cells grown in the presence of 10 µM plumbagin for 14 h were either serially four-fold diluted, spotted onto YES plates, and incubated for 3 days and photographed when colonies appeared (C) or observed under microscope (D). (E) After the treatment in D, mRNA was prepared and the levels of mRNAs of its3 and Act1 were measured using RT-PCR analysis. RT-PCR analysis was performed according to the following procedures. Total RNA was isolated by lysis with glass beads in the presence of Accuzol (BIONEER). RT-PCR amplification of total RNA (1 µg/reaction) was performed using *its3* -specific primers (forward, 5′-GATGGCATTCCCCCCGATATTG-3; reverse, 5′-TCGTCGAGTTCCCTTCCTAGGG-3′) and *act1*-specific primers (forward, 5′- CACCCTTGCTTGTTGACTGAGGC-3; reverse, 5′-AGCTTCAGGGGCACGGAAACGC-3′) in a 20 -µl reaction using the Accupower RT/PCR PreMix (Bioneer, Daejeon, Korea). PCR amplification was performed as follows: one cycle at 42°C for 1 h, 94°C for 5 min, 30 cycles at 94°C for 30 sec, 55°C for 30 sec, 72°C for 30 sec, and one final extension cycle at 72°C for 5 min. PCR reaction products were analyzed by agarose gel electrophoresis.

We next tried to define the molecular targets of plumbagin to understand the detailed mechanisms for ROS generation. For this purpose, we investigated DIH by plumbagin in various *S. pombe* heterozygous deletion mutants (Table 1). Interestingly, among the various mutants that were treated with different doses of plumbagin for 14 h, the most substantive DIH was observed in its3-deleted heterozygous mutant cells (Figure 3); its3 is a type of PI5K in *S. pombe*. Therefore, we further investigated the functional role of its3 in the growth-inhibitory activity of plumbagin in *S. pombe*. When compared to wild-type cells, plumbagin-induced growth inhibition was dramatically increased in the its3-deleted heterozygous mutant in a liquid culture assay (Figure 4A & B), which was confirmed in a plot assay on agar plate (Figure 4C) and in microscopic observation (Figure 4D). Targeted gene deletion of its3 in haploid *S. pombe* cells abrogated colony formation, implying that its3 is an essential gene for cell survival of *S. pombe* and that targeted inhibition of its3 by plumbagin might contribute to the observed cytotoxicity.

**Table 1:** *S. pombe* heterozygous deletion mutants for drug-induced haploinsufficiency.

Gene Name	Systemic ID	Gene Function
Suc1	SPBC1734.14c	Cyclin-dependent protein kinase regulatory subunit
Cut12	SPBC649.05	Spindle pole body protein
Act1	SPBC32H8.12c	Actin
Tif45	SPAC16E8.15	Translation initiation factor eIF4E, 4F complex subunit
Cdc2	SPBC11B10.09	Cyclin-dependent protein kinase
Tif35	SPBC18H10.03	Translation initiation factor eIF3g
Sum1	SPAC4D7.05	Translation initiation factor eIF3i
Tuf1	SPBC9B6.04c	Mitochondrial translation elongation factor EF-Tu
Tor2	SPBC216.07c	Phosphatidylinositol kinase Tor2
Its3	SPAC19G12.14	1-Phosphatidylinositol-4-phosphate 5-kinase

**Figure 5:**
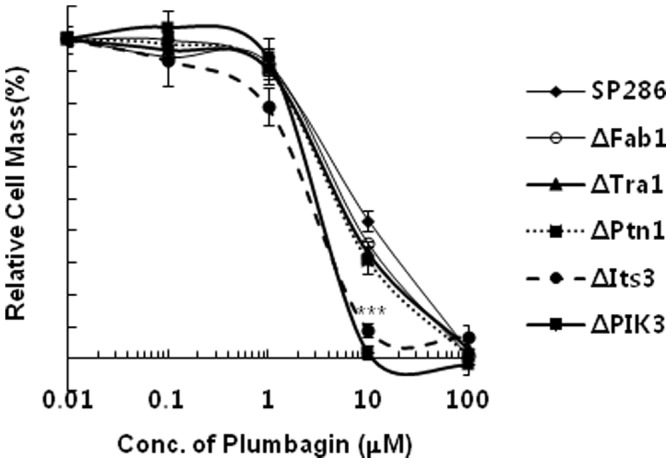
Comparative analysis of DIH by plumbagin between an its3-deletion mutant and a PI3K-deletion mutant. All *S. pombe* cells, including the wild-type and mutants indicated, were treated under the same conditions as in Figure 3 with various doses of plumbagin for 14 h, and the relative cell mass was analyzed by measuring OD_600_. Data represent the mean ± standard error (n = 3).

There was no change in the level of its3 mRNA by plumbagin in wild-type *S. pombe,* implying that plumbagin might inhibit its3 at protein levels (Figure 4E). Because PI3K is a known target of plumbagin in human breast cancer cells [14], we next compared the effects of its3 deletion on the DIH with that of PI3K deletion. For this purpose, we investigated the DIH in a PIK3-deleted mutant because PIK3 is an *S. pombe* ortholog of human PI3K; we also analyzed other mutants that contain only one copy of other related PIKs such as FAb1 (1-phosphatidylinositol-3-phosphate 5-kinase), Tra1 (phosphatidylinositol kinase), and Ptn1 (phosphatidylinositol-3, 4, 5-trisphosphate 3-phosphatase). Interestingly, DIH in its3-deleted mutants was comparable to that in PIK3-deleted cells. However, DIH was not observed in the other deletion mutants (i.e., FAb1, Tra1 and Ptn1), implying that its3 is a promising and selective target of plumbagin for cytotoxicity (Figure 5).

### Its3 Deletion Potentiates ROS Generation by Plumbagin in *S. pombe*

We next investigated the functional role of its3 in plumbagin-mediated ROS generation. To this end, we first tested whether its3 deletion affects the level of ROS in normal conditions. The basal level of ROS was not changed significantly in DHE staining and fluorescence microscopy observations in its3-deleted cells compared to those of wild-type cells (Figure S5).

**Figure 6:**
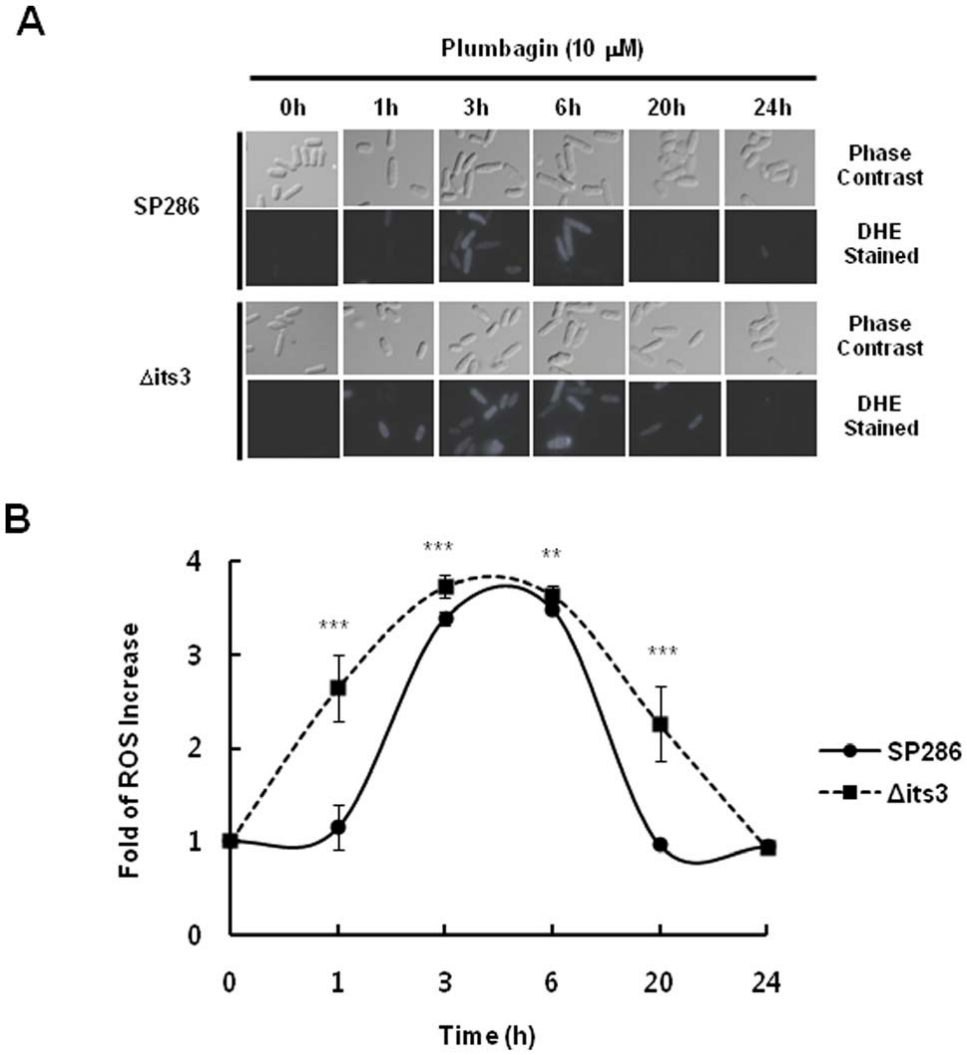
Its *3*-dependent ROS generation by plumbagin in *S. pombe*. (A) Wild-type and the its3-deletion mutant were treated with 10 µM plumbagin and incubated for each time indicated. Then, they were stained with 4 µM DHE for 30 min, and the level of ROS was observed by fluorescence microscopy. (B) The level of ROS in the treated cells was measured by fluorescence microscopy as indicated in Materials and Methods. Data represent the mean ± standard error (n = 3). *P* values compared with wild type (SP286) cells.

When we investigated the changes of ROS level in the presence of plumbagin, ROS increase was prominent in 3–6 h in wild-type cells (SP286) (Figure 6, upper lane). Interestingly, the ROS increase by plumbagin was greater and more prolonged in its3-deleted heterozygous mutants compared to that in wild-type cells (Figure 6, bottom lane). This result suggested that its3 contributes to ROS generation as a molecular target of plumbagin for cytotoxicity in *S. pombe* cells.

### Plumbagin Decreases PI-5 kinase-1B Level, and siRNA-mediated PI-5 Kinase-1B Knockdown Decreases MCF-7 cell Viability

**Figure 7:**
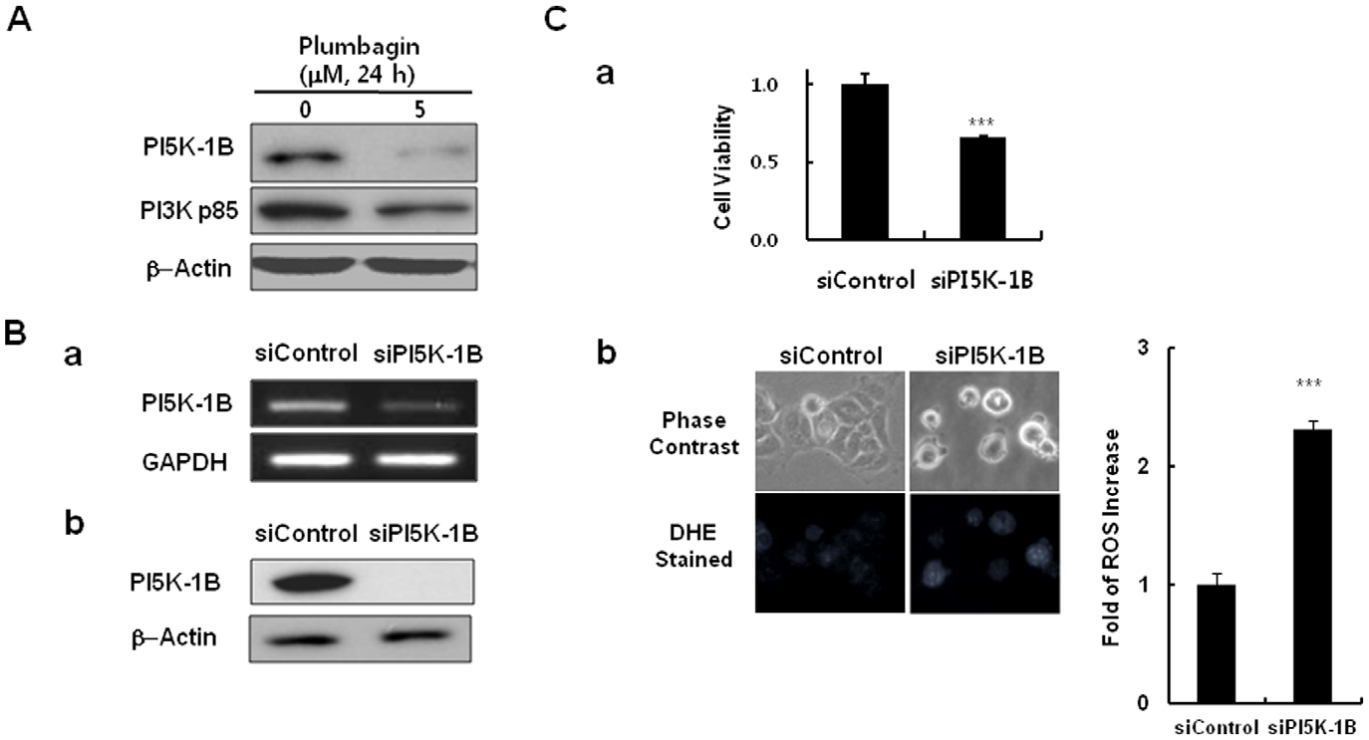
Downregulation of PI5K-1B by plumbagin and functional role of PI5K-1B for cell viability and ROS generation in MCF-7 human breast cancer cells. (A) MCF-7 cells were cultured in a 6-well plate as described in Materials and Methods and then either left untreated or treated with 5 µM plumbagin for 24 h. Then, cells were harvested, and the levels of proteins in the cell lysates were analyzed by western blotting (panel A). (B) PI5K-1B knockdown in MCF-7 cancer cells was carried out a described in Materials and Methods using PI5K-1B-specific siRNA (5′-GUCCUCAAUUAGCCAGGAA(dTdT)-3′) or a control siRNA (5′-CUUACGCUGAGUACUUCGA(dTdT)-3′) for 48 h. Then, knockdown of PI5K-1B was validated by either RT-PCR (panel Ba) or western blotting (panel Bb) as described in Materials and Methods. (C) Cell viability after siRNA transfection for 48 h was measured by the WST-1 assay. Data represent the mean ± standard error (n = 3) (panel Ca). ***P<0.001 compared with the untreated samples. The cells after siRNA transfection for 48 h were stained with 4 µM DHE for 30 min, and then the ROS level was observed using a fluorescence microscope (excitation 518 nm, emission 605 nm) (panel Cb).

Because the above data implicated its3 as a molecular target of plumbagin in yeast, we performed a target validation study in human cancer cells. First, we found that PI5K-1B is a human ortholog of *S. pombe* its3 through protein sequence comparative analysis using an NCBI BLAST search, and therefore we investigated whether PI5K-1B is regulated by plumbagin in MCF-7 cells. Interestingly, the level of PI5K-1B decreased significantly in the treatment with 5 µM plumbagin for 24 h. PI3K p85, which is a molecular target already proved, was also decreased in our test (Figure 7A). The level of PI5K-1B was not significantly decreased in other type of cancers such as HeLa human cervical cancer cells and A549 human lung carcinoma cells (Figure S6), which were less sensitive to plumbagin than MCF-7 breast cancer cells, implying that the sensitivity of MCF-7 breast cancer cells to plumbagin might be possibly linked to the regulation of PI5K-1B by the agent. This result clearly showed that plumbagin inhibits PI5K-1B in MCF-7 human breast cancers as a promising molecular target of this agent.

**Figure 8:**
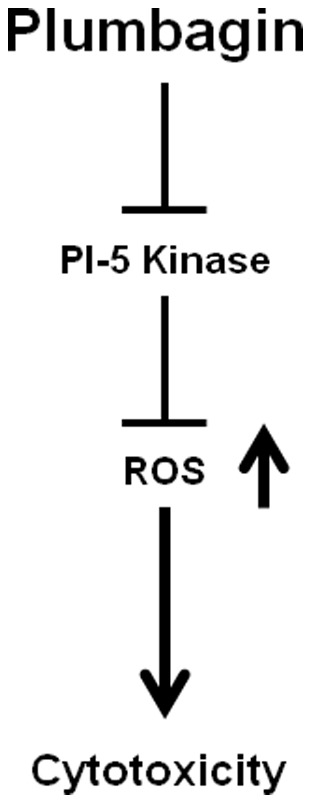
Diagram depicting mode of action of plumbagin.

Finally, we examined the functional role of the decrease of PI5K-1B by plumbagin in cancer cells. MCF-7 cells were transfected with 20 nM PI5K-1B-selective siRNA or with control siRNA for 48 h, and cell viability was measured using the WST-1 assay. Upon knockdown of PI5K-1B, as revealed by RT-PCR and western blotting (Figure 7Ba & Bb), the viability of siPI5K-1B-treated cells decreased significantly by 34% compared with control cells (Figure 7Ca), supporting the critical role of PI5K-1B for cell viability in cancer cells. Especially, PI5K-1B knockdown induced a dramatic increase in ROS in treated cells compared to controls (Figure 7Cb), suggesting that PI5K-1B controls ROS generation in human cancer cells (Figure 8). These results indicated that PI5K-1B might play a crucial role as a molecular target of the natural anticancer agent, plumbagin, for ROS-mediated cytotoxicity in MCF-7 cells.

## Discussion

Precise drug target identification is a critical issue for increasing the success rate of drug discovery as well as understanding the mode-of-action of drug candidates, considering that about half of the drug R&D failures are caused by problems such as toxicity and lack of efficacy of drug candidates, which could be traced and resolved by defining their molecular targets.

In our present study in which we elucidated drug targets of plumbagin using DIH in fission yeast *S. pombe*, which is considered a superior model organism for mammalian cells because *S. pombe* has very similar biological patterns to human cancer cells, plumbagin showed potent growth inhibitory activity (GI_50_ = 10 µM; Figure 2A) and cell elongation and septum formation (Figure 2B), a representative of G2-M arrest in human cancers [14]. Notably, plumbagin induced ROS generation in the yeast cells (Figure 2C). Furthermore, prior treatment of an ROS scavenger NAC protected the growth inhibition of *S. pombe* by plumbagin (Figure 2D), supporting the pivotal role of ROS in plumbagin-induced cytotoxicity. These results provide the underlying basis for drug target screening in *S. pombe* heterozygous deletion mutants to understand molecular insights for plumbagin-induced ROS generation.

Through drug target screening using DIH in various *S. pombe* heterozygous deletion mutants, we fortunately found that a prominent DIH was occurred in its3-deleted mutants (Figure 3, 4, and Table 1). In a comparison analysis among various PIKs in *S. pombe,* the potency of DIH in a PI5K its3-deleted mutant was comparable to that in PIK*3* (*S. pombe* ortholog of human PI3K)-deleted mutant, but not found in other types of PIK-deletion mutants such as Fab1, Tra1, and Ptn1 (Figure 5). Considering that the PI3K subunit p85, NF-kB and Bcl2 are inhibited by plumbagin in human breast cancer cells [14], [15], this result suggests that its3 is a new molecular target of plumbagin and possibly contributes to the cytotoxic activity of this agent in human cancers. Notably, we also suggest a functional role for its3 in plumbagin cytotoxicity. Interestingly, the increase of ROS by plumbagin was more potent and prolonged in the its3-deleted mutant (Figure 6). Until now, there has been no report about regulation of PI5K by plumbagin. Our report is the first to demonstrate that PI5K controls ROS generation as a molecular target of plumbagin. These results strongly suggest that targeted inhibition of PI5K by plumbagin plays a major role in ROS generation related to cytotoxicity.

Furthermore, we validated the identified target of plumbagin from *S. pombe* in human cancer cells. In the treatment to MCF7 human breast cancer cells, plumbagin showed the potent cytotoxicity in the treated cells (Figure 1) and substantially decreased the level of PI5K-1B, which is a human ortholog of *S. pombe* its3 (Figure 7A), showing that PI5K-1B is a molecular target of plumbagin in MCF7 human breast cancer cells. Furthermore, knockdown of PI5K-1B using siPI5K-1B resulted in a substantial decrease in cancer cell viability, indicating that PI5K-1B plays a crucial role in the anticancer activity as a molecular target of plumbagin (Figure 7Ca). More interestingly, we also found that PI5K-1B controls ROS generation in the cancer cells; PI5K-1B knockdown dramatically increased the intracellular ROS level compared to control (Figure 7Cb).

Even though the functional roles of PI3K as an anticancer therapeutic target are well known [23], [24], the functional roles of PI5K in this respect have not been reported. *S. pombe* its3, which is an ortholog of human PI5K, is an essential gene for cell survival, and therefore only diploid its3-deleted heterozygous mutant, but not haploid its3-deleted mutant, survived. At this time, we do not know the detailed mechanism by which PI5K-1B is decreased by plumbagin. It might be a result, at least in part, of the inhibition of protein synthesis by plumbagin because translation initiation factors such as Tif35 (eIF3i in human) and Sum1 (eIF3g in human) and translation elongation factor Tuf1 (EF-Tu) were shown to be partially inhibited by plumbagin in our DIH study (Figure 3). Interestingly, previous report have shown that plumbagin inhibits DNA binding of transcription factors (TFs) and TF-regulated gene expression [9]. Furthermore, the mechanism may also be derived by the inhibition of Ras-family proteins such as Ypt2 and Ypt5, which are critical regulators of gene expression for mitogenic signaling activation, and were shown to be dramatically inhibited by plumbagin in our DIH study in *S. pombe* (Figure S7 & Table S1). However, we do not know the relevance between these factors and the regulation of PI5K-1B by plumbagin, an issue that needs to be solved in further studies.

Taken together, this is the first report showing the functional relevance of a PI-5 kinase as a molecular target of anticancer agents. It is well established that ROS act as cytotoxic mediators of many anticancer agents [25], [26], [27]. However, its detailed molecular mechanisms have not been defined, and, considering that plumbagin inhibits Ras-family G-proteins Ypt2/5, ERK pathway and Nox-1 group enzymes could be possibly involved in the induction of reactive oxygen species [28], which need to be solved in next study. Therefore, our observation that plumbagin decreases the level of PI5K-1B, thereby leading to ROS generation for cytotoxicity in human breast cancer cells, may shed light on the understanding of the molecular basis for ROS generation and provide therapeutic clues for developing more effective anticancer therapeutics whose effects are mediated by ROS generation. This report also shows that drug target identification using an *S. pombe* heterozygous deletion mutant library is a very valuable tool for both drug target identification and mode-of-action studies of drug candidates.

## Supporting Information

Figure S1 **Cytotoxic effects of plumbagin on various human cancer cells.** Cells were plated onto 6-well plates at 2×10^4^ cells/well. The following day, cells were treated with plumbagin and incubated for 24 h. Then, cells were observed.
(TIF)

Figure S2 **Cytotoxic effects of plumbagin on MCF-7 human breast cancer cells and A549 human lung cancer cells.** Cells were plated onto 6-well plates at 2×10^4^ cells/well. The following day, cells were treated with 10 µM of plumbagin, and incubated for 24 h. Then, cells were photographed (A). The viability of the treated cells in was measured with the WST-1 assay (B). Data represent the mean ± standard error (n = 3).
(TIF)

Figure S3 **Cytotoxic effects of plumbagin on A549 human lung cancer cells in an ROS-dependent manner.** A549 cells were plated onto 6-well plates at 2×10^4^ cells/well. The following day, cells were treated with 10 µM of plumbagin in the presence or absence of 2 mM NAC, a ROS scavenger, and incubated for 24 h. Then, cells were photographed (A). The viability of the treated cells in was measured with the WST-1 assay (B). Data represent the mean ± standard error (n = 3).
(TIF)

Figure S4 **The effects of plumbagin and NAC on ROS generation in wild-type** ***S. pombe.***
(TIF)

Figure S5 **Basal level of ROS in wild-type S. pombe or its3-, or PIK3-deleted mutants.**
(TIF)

Figure S6 **Western blot analysis of PIK-1B in A549 (lung cancer) and HeLa (cervical cancer) cells after treatments of 10 µM plumbagin for 24 h?**
(TIF)

Figure S7 ***S. pombe*** **genome-wide drug-induced haploinsufficiency screens for defining targets of plumbagin in 351 essential genes.** After the treatment of 10 µM of plumbagin to 351 essential genes-deletion mutants for 14 h, the fitness values of each mutant among 351 essential genes, which was described in more details in Supple.-Table 1, were plotted.
(TIF)

Table S1 **Results of DIH screenings in** ***S. pombe*** **heterozygous deletion mutants of 351 essential genes.** Top 20 s of the potential drug targets in the DIH screening was listed.
(TIF)
