# Supplementary figures and images for: The Natural Anticancer Agent Plumbagin Induces Potent Cytotoxicity in MCF-7 Human Breast Cancer Cells by Inhibiting a PI-5 Kinase for ROS Generation
Source: PLoS One. 2012 Sep 13;7(9):e45023. doi: 10.1371/journal.pone.0045023 (PMC3441601; doi:10.1371/journal.pone.0045023)

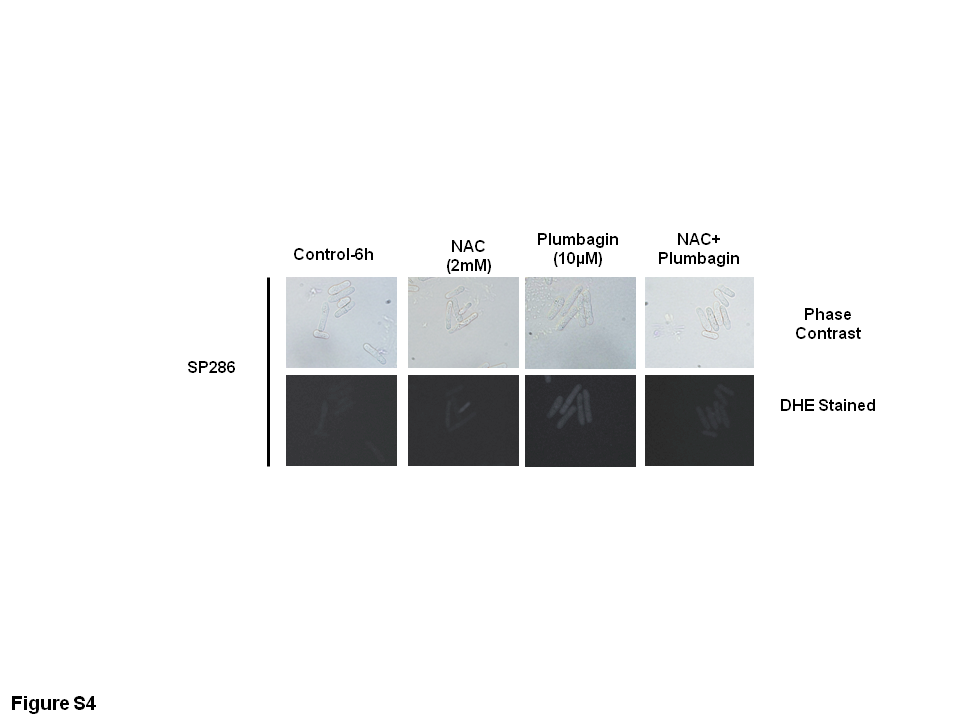

Supplement: Figure S4 — The effects of plumbagin and NAC on ROS generation in wild-type S. pombe. (TIF) [file pone.0045023.s004.tif]

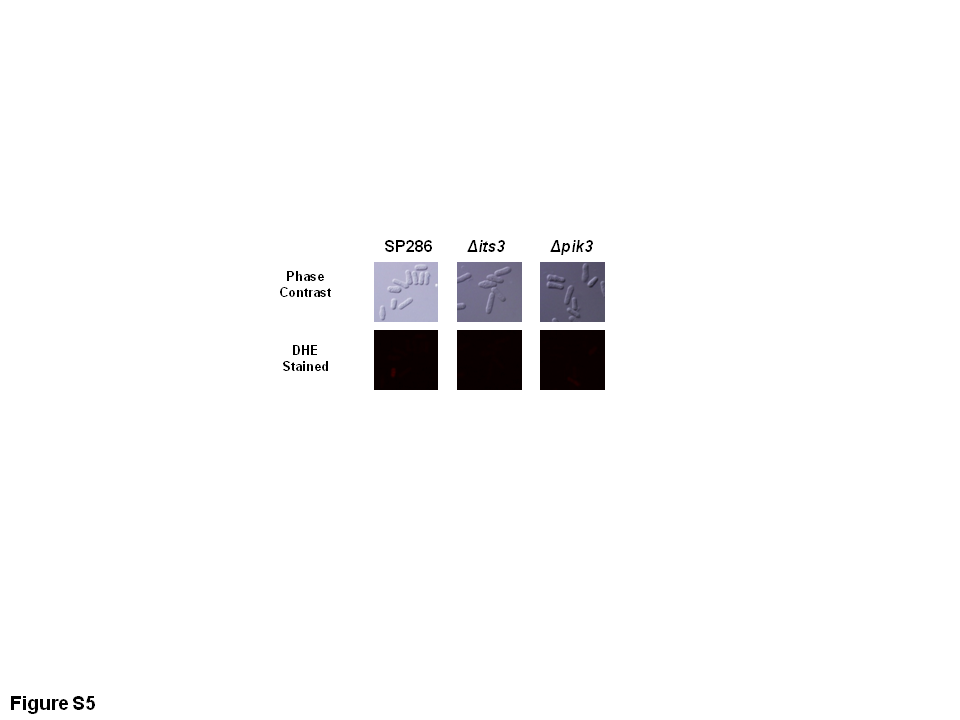

Supplement: Figure S5 — Basal level of ROS in wild-type S. pombe or its3-, or PIK3-deleted mutants. (TIF) [file pone.0045023.s005.tif]

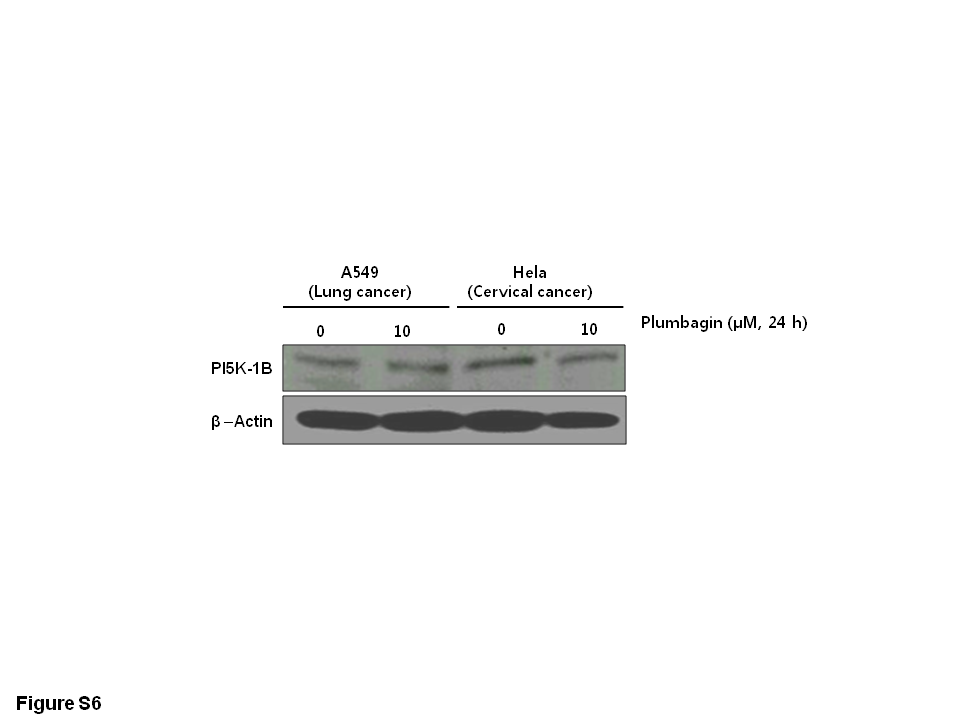

Supplement: Figure S6 — Western blot analysis of PIK-1B in A549 (lung cancer) and HeLa (cervical cancer) cells after treatments of 10 µM plumbagin for 24 h? (TIF) [file pone.0045023.s006.tif]
